# *Noctiluca scintillans* Bloom Reshapes Microbial Community Structure, Interaction Networks, and Metabolism Patterns in Qinhuangdao Coastal Waters, China

**DOI:** 10.3390/microorganisms13081959

**Published:** 2025-08-21

**Authors:** Yibo Wang, Min Zhou, Xinru Yue, Yang Chen, Du Su, Zhiliang Liu

**Affiliations:** 1 Research Center for Marine Science, Hebei Normal University of Science and Technology, Qinhuangdao 066004, China; wyb4044@hevttc.edu.cn (Y.W.);; 2 Hebei Key Laboratory of Ocean Dynamics, Resources and Environments, Qinhuangdao 066004, China

**Keywords:** *Noctiluca scintillans* bloom, microbial community, environmental drivers, co-occurrence network, metabolism function

## Abstract

The coastal waters of Qinhuangdao are a major hotspot for harmful algal blooms (HABs) in the Bohai Sea, with *Noctiluca scintillans* being one of the primary algal species responsible for these events. A comprehensive understanding of the microbial community structure and functional responses to *N. scintillans* bloom events is crucial for elucidating their underlying mechanisms and ecological impacts. This study investigated the microbial community dynamics, metabolic shifts, and the environmental drivers associated with a *N. scintillans* bloom in the coastal waters of Qinhuangdao, China, using high-throughput sequencing of 16S and 18S rRNA genes, co-occurrence network analysis, and metabolic pathway prediction. The results revealed that the proliferation of autotrophic phytoplankton, such as *Minutocellus* spp., likely provided a nutritional foundation and favorable conditions for the *N. scintillans* bloom. The bloom significantly altered the community structures of prokaryotes and microeukaryotes, resulting in significantly lower α-diversity indices in the blooming region (BR) compared to the non-blooming region (NR). Co-occurrence network analyses demonstrated reduced network complexity and stability in the BR, with keystone taxa primarily belonging to Flavobacteriaceae and Rhodobacteraceae. Furthermore, the community structures of both prokaryotes and microeukaryotes correlated with multiple environmental factors, particularly elevated levels of NH_4_^+^-N and PO_4_^3−^-P. Metabolic predictions indicated enhanced anaerobic respiration, fatty acid degradation, and nitrogen assimilation pathways, suggesting microbial adaptation to bloom-induced localized hypoxia and high organic matter. Notably, ammonia assimilation was upregulated, likely as a detoxification strategy. Additionally, carbon flux was redirected through the methylmalonyl-CoA pathway and pyruvate-malate shuttle to compensate for partial TCA cycle downregulation, maintaining energy balance under oxygen-limited conditions. This study elucidates the interplay between *N. scintillans* blooms, microbial interactions, and functional adaptations, providing insights for HAB prediction and management in coastal ecosystems.

## 1. Introduction

Marine ecosystems, especially coastal waters, are currently facing unprecedented challenges, such as global warming, nutrient pollution, and overfishing, which directly threaten ecosystem health [1,2]. Among these ecological disasters, harmful algal blooms (HABs) have gained significant attention due to their detrimental effects on marine ecosystems and human health. HABs are ecological anomalies that cause discoloration of seawater due to overpopulation of certain plankton under specific physicochemical factors and nutrient conditions, resulting in abnormal ecosystem structure and function [3]. It is estimated that no less than 2000 cases of poisoning due to algal toxins occur globally each year, with an economic loss of about 4 billion US dollars [4,5]. In China, the direct economic losses attributed to phycotoxins from 1977 to 2019 reached 5.9 billion yuan (about 0.87 billion US dollars), with actual losses likely being even higher [6]. Thus, it is crucial to enhance our understanding of the ecological dynamics surrounding harmful algae to effectively manage and control this phenomenon.

The formation, development, maintenance and termination of HABs are complex processes influenced by both abiotic and biotic factors. Abiotic factors include environmental conditions and nutrient availability, while biotic factors encompass grazing, pathogenicity and parasitism [7,8,9,10]. Among these biotic factors, microorganisms play a crucial role in HAB occurrence and development. They form complex ecological networks through various interactions, such as symbiotic mutualism [11], algicidal effects [8], allelopathic interactions [12], and parasitism [9]. These interactions support various ecosystem functions, including matter and energy flow and ecosystem stability [13,14]. Moreover, microorganisms affect the structure of microbial food webs through their participation in biogeochemical cycles, competing with algae for ecological niches [15,16]. Conversely, HABs can also affect microbial community structures and food webs [17,18]. Understanding the complex interactions among microorganisms and between microorganisms and the causative algal species of HABs is essential for elucidating the mechanisms that underlie HAB occurrences.

The Qinhuangdao coastal waters are a major hotspot for HABs in the Bohai Sea, China, with frequent blooms causing substantial economic losses to local fisheries and tourism [19,20]. One of the main algal species responsible for these blooms is the heterotrophic dinoflagellate *Noctiluca scintillans* [19], which is known for emitting blue fluorescence when disturbed. *N. scintillans* thrives in relatively low water temperatures (10–25 °C) [21] and typically causes HABs in the Qinhuangdao coastal waters most frequently during April–June and September–October [19]. Although *N. scintillans* does not produce toxins, it can secrete mucus and release ammonia, which can adversely affect the marine environment and organisms [2,22]. Research on outbreak mechanisms of *N. scintillans* blooms primarily focuses on nutrient supply [23] and hydrodynamic processes [24]. The dynamics of *N. scintillans* blooms correlate with phosphate and silicate levels, while precipitation, hydrodynamics, temperature, and food availability influence their spatial and temporal distribution along the Chinese coast [25]. Conversely, *N. scintillans* also enhances nutrient regeneration, supplying significant amounts of nitrogen and phosphorus to phytoplankton in the upper water layers [26,27].

Recently, an increasing number of studies have focused on microbial community composition and function during *N. scintillans* blooms, showing that structural changes and functional differences in microbial communities can respond to and affect the *N. scintillans* blooms [18,28,29]. For instance, Xia et al. reported that *N. scintillans*-associated bacterial community is dominated by Vibrionaceae during the bloom stage and exhibits significant downregulation of genes related to complex carbohydrate metabolism, while genes related to glucose transport and utilization are upregulated compared to the non-bloom stage [28]. Similarly, Zhou et al. suggested that microbial community composition and function display significant temporal heterogeneity at different stages of *N. scintillans* blooms, with phycosphere microorganisms enhancing organic carbon decomposition capacity, altering nitrogen assimilation rates, and influencing sulfur/phosphorus turnover efficiency and iron budget balance during HAB processes [18]. These findings indicate that structural and functional changes in microbial communities can respond to and affect *N. scintillans* blooms, yet the underlying mechanisms remain poorly understood. Therefore, in-depth investigations into the role of microorganisms in *N. scintillans* blooms are essential for providing valuable insights that could aid in the regulation of HAB dynamics in marine environments.

Through integrated analysis of 16S/18S rRNA gene high-throughput sequencing data, microbial co-occurrence networks, and gene predictions, this study systematically investigates: (1) the environmental and biological drivers underlying the *N. scintillans* bloom formation, and (2) bloom-induced shifts in microbial community structure and function. Studying these issues will enhance our understanding of the impacts of HABs on the function and stability of marine ecosystems and provide essential information for the prediction and management of HABs.

## 2. Materials and Methods

### 2.1. Study Area and Sampling Sites

**Figure 1:**
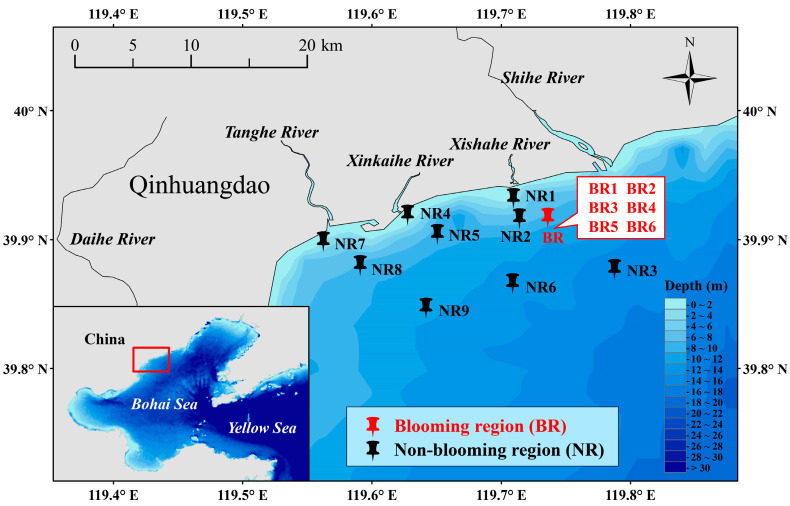
Location of sampling region and sites. Since the six sites in the blooming region (BR) were randomly selected and located close to each other, only a representative central location is marked on the map.

**Figure 2:**
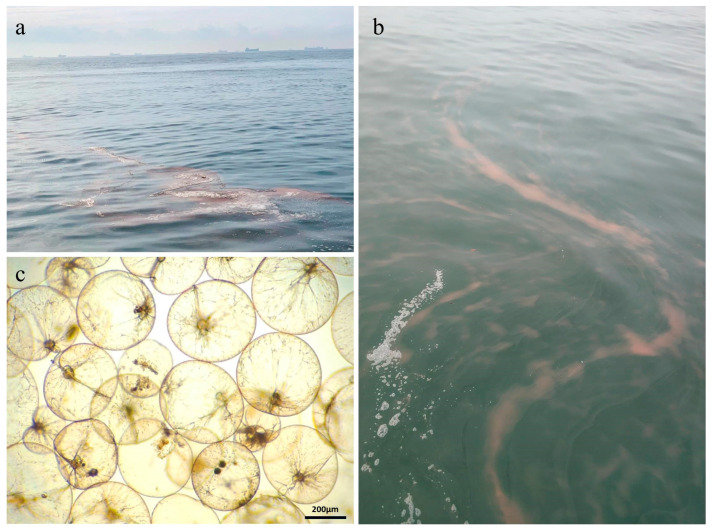
Images of the *Noctiluca scintillans* bloom: (**a**) Blooming scene; (**b**) Flocculent aggregates formation; (**c**) Microscopic cell morphology.

The study area is located in the coastal waters of Qinhuangdao, China. The survey was conducted on 28 September 2021. Grid sampling was conducted in the area where the *Noctiluca scintillans* bloom was absent (119°47′33″ E–119°33′44″ E, 39°57′17″ N–39°50′49″ N), designated as the non-blooming region (NR) below. Nine grid sites (NR1-NR9) were selected within the NR for sampling. The central location of the *N. scintillans* bloom was roughly 119°44′09″ E, 39°55′00″ N, covering an area of nearly 100 m^2^ (Figure 1 and Figure 2). Its distribution is influenced by wind, tides, and currents, making precise area measurement and grid sampling challenging. Additionally, flocculent cell clumping results in patchy density. Therefore, six randomized sites (BR1–BR6) were sampled within the blooming region (BR) to statistically represent the bloom’s patchiness. Seawater samples were collected from the surface, middle, and bottom layers at each site in both the BR and the NR (Table S1).

### 2.2. Sampling and Laboratory Analysis

Depth, temperature, salinity, pH, and dissolved oxygen (DO) were measured in situ using a calibrated Conductivity-Temperature-Depth (CTD) system (SeaBird, Loveland, CO, USA). Seawater samples were collected from surface, middle and bottom layers at each site using an SBE 32 Water Sampler (SeaBird, Loveland, CO, USA). In total, 45 seawater samples were collected. For each sample, 1 L of seawater was filtered through a 200 μm mesh sieve to remove larger zooplankton. The water was then passed through a 0.2 μm polycarbonate filter membrane (Millipore, Burlington, MA, USA). The filters were stored in liquid nitrogen during the cruise and subsequently transferred to a −80 °C deep freezer in the laboratory for future DNA extraction. Approximately 100 mL of filtered seawater was collected in 100 mL polycarbonate bottles and stored at −20 °C for nutrient concentration analysis. Additionally, 500 mL of seawater was sampled and filtered using a 0.7 μm GF/F filter membrane (Whatman, Maidstone, UK) and preserved at −20 °C for chlorophyll *a* concentration analysis. Nutrient concentrations were determined using a continuous flow analyzer (QuAAtro39, SEAL Analytical, Norderstedt, Germany). Standard solutions with filtered seawater matching the salinity of the samples were prepared. Prior to analysis, samples were equilibrated to room temperature, and ammonium samples were covered with sealing film to reduce potential volatilization interference. The calibration curve exhibited a correlation coefficient of ≥0.9999, and samples were analyzed in ascending order of concentration. Chlorophyll *a* concentration was determined using a 10-AU fluorometer (Turner Designs, San Jose, CA, USA) after 24 h extraction in 90% acetone at −20 °C. Another 500 mL of seawater was collected from each depth layer and fixed in polyethylene bottles with formaldehyde (final concentration 5%) for microscopy analysis. After 24–48 h of sedimentation, the fixed seawater samples were concentrated to a final volume of 50–250 mL. *N. scintillans* cells were enumerated in a 500-μL counting chamber by performing ten replicate counts under the UB203i inverted microscope (UOP Photoelectric Technology, Chongqing, China). The abundance was calculated based on the counted cell numbers and the corresponding concentration factor. The occurrence of the *N. scintillans* bloom was confirmed based on the density threshold (3 × 10^3^ cells/L) as specified in the Technical Regulations of Red Tide Monitoring (HY/T 069-2005).

### 2.3. DNA Extraction, PCR and Sequencing

Genomic DNA from microbial samples was extracted from the filters using the FastDNA^®^ Spin Kit (MP Biomedicals, Solon, OH, USA) according to the manufacturer’s instructions. The extracted DNA was quantified using an enzyme marker (GeneCompang Limited, synergy HTX, Singapore), and its integrity was checked by 1.8% agarose gel electrophoresis. Subsequently, the hypervariable V4 region of prokaryotic 16S rRNA genes and V9 region of eukaryotic 18S rRNA genes were amplified, since these regions are widely adopted in microbial ecology due to their high taxonomic resolution and compatibility with Illumina platforms [30,31]. The V4 region of the prokaryotic 16S rRNA gene was amplified with the primer pair 515F [30] and 806R [32], while the V9 region of the eukaryotic 18S rRNA gene was amplified using the primer pair 1380F [31] and 1510R [31]. All PCR reactions were performed in a 10 μL reaction system, consisting of template DNA (2.5–4 ng), KOD FX Neo Buffer (5 μL), dNTP (2 mM each) 2 μL, and KOD FX Neo 0.2 μL. The PCR cycling conditions included an initial denaturation step at 95 °C for 5 min, followed by 25 cycles of 95 °C for 30 s, 50 °C for 30 s, and 72 °C for 40 s, concluding with a final extension at 72 °C for 7 min, and then holding at 4 °C. The amplification products were analyzed using 1.8% agarose gel electrophoresis, and the target fragments were subsequently recovered using the Monarch DNA Gel Recovery Kit. Finally, the purified amplicons were pooled and sent for high-throughput paired-end sequencing on the Illumina HiSeq 2500 platform at Biomarker Technologies Corporation in Beijing, China.

### 2.4. Bioinformatics Analysis

The raw paired-end reads were processed in R (version 4.3.1) using the DADA2 package in accordance with the DADA2 Pipeline Tutorial (version 1.16) (https://benjjneb.github.io/dada2/tutorial.html, accessed on 1 July 2024). The 16S and 18S rRNA gene sequences were annotated using the Silva database (version 132, http://www.arb-silva.de, accessed on 3 July 2024) and the PR2 database (version 5.0.0, https://github.com/pr2database, accessed on 3 July 2024), respectively. This process generated amplicon sequence variant (ASV) abundance tables and taxonomic species annotation tables. ASVs originating from chloroplasts or mitochondria in the prokaryotic dataset were removed using the phyloseq package. Similarly, ASVs in the eukaryotic dataset that were assigned to Metazoa and plants (i.e., Rhodophyta, Streptophyta, Trebouxiophyceae, and Ulvophyceae) were also removed. Additionally, ASVs that were unclassified at the phylum level were removed prior to further analysis. To ensure comparability among samples in terms of sequencing depth, the vegan package was used to normalize the ASV abundance table data based on the minimum sequencing depth across samples.

### 2.5. Data Processing and Community Structure Analysis

Data processing for microbial community structure and co-occurrence networks was conducted using R software (version 4.3.1). The analysis of α- and β-diversity of microbial communities across different regions was performed using the ggplot2 (version 3.4.2) and vegan (version 2.5-7) packages. Specifically, the α-diversity indices included the Sobs, Shannon, and Pielou’s indices [33]. Sobs index assesses the species richness within a community, while Pielou’s index evaluates the evenness of species distribution. In contrast, Shannon index considers both species richness and evenness. The β-diversity was quantified through Principal Coordinate Analysis (PCoA) using Bray–Curtis distance to evaluate the similarity between microbial communities across samples. Additionally, the statistical significance of these differences was validated using the Wilcoxon rank-sum test and Permutational Multivariate Analysis of Variance (PERMANOVA). Venn diagrams were created using the VennDiagram (version 1.7.3) package to analyze shared and unique ASVs between regions, while the relative abundance at the genus level was assessed with the ggplot2 package. The Wilcoxon rank-sum test was employed to test for significant differences in dominant species between regions and statistical significance was set at *p* < 0.05. Redundancy Analysis (RDA) was performed using the vegan package based on Hellinger-transformed abundance data and log_10_(x + 1)-transformed environmental data. The mean substitution method was used to handle the missing data in nutrient concentrations. Subsequently, environmental variables with VIF > 10 were excluded from RDA to avoid the collinearity problem, and the key environmental factors (*p* < 0.05) were identified by forward selection (FS) using the ordiR2step function in the vegan package. Then, ordination plots of environmental factors with prokaryotes and microeukaryotes were generated using the ggplot2 package.

### 2.6. Co-Occurrence Network Analysis

Co-occurrence networks for prokaryotes and microeukaryotes were constructed using the Hmisc (version 5.0.1) and igraph (version 1.2.11) packages. First, surface sample data from the BR and the NR were extracted from the ASV table for the experimental data. The data were then simplified by retaining only those ASVs with a relative abundance greater than 0.01% and present in more than 20% of the samples [34]. The association matrix was computed based on Spearman correlations, incorporating only the correlations deemed robust (|*R*| > 0.6) and statistically significant (*p* < 0.05) for network analysis, which was then exported as GraphML format. The fast greedy algorithm was utilized to identify modules, and information regarding nodes and edges, including node and edge weights, and module affiliation of nodes, was also incorporated into the GraphML dataset. The network was visualized using Gephi (v0.10.1). Node attributes (average degree, betweenness centrality), network topological properties (number of nodes, number of edges, average path length, clustering coefficient), and modular properties (modularity, number of modules) were computed using the igraph package.

### 2.7. Gene Prediction Analysis

To characterize microbial metabolic functional shifts during the algal bloom, metabolic pathways were predicted from 16S rRNA gene sequences using PICRUSt2 (v2.6.2) with the MetaCyc database as reference. Differential abundance analysis of metabolic pathways, KEGG orthologs (KOs), and enzyme-coding genes was performed, along with identification of key functional taxa, using R packages dplyr, tidyr, and ggplot2. This study primarily focused genes classified under “metabolism” at the KEGG level 1. Differential expression patterns (up-/down-regulation) were determined based on Wilcoxon rank-sum testing of gene relative abundance, with a significance threshold of *p* < 0.05.

## 3. Results

### 3.1. Noctiluca scintillans Density and Environmental Conditions

In the surface samples of the BR, the maximum density of *N. scintillans* was 2.8 × 10^4^ cells/L, exceeding the established bloom threshold of 3 × 10^3^ cells/L. Based on *N. scintillans* density and non-depleted dissolved oxygen levels in the BR, the sampling likely occurred during the developmental stage of the bloom. Significant differences in physicochemical factors were observed between the BR and NR across water layers (*p* < 0.05), except for the middle layer where no significant differences occurred (*p* > 0.05; Table 1). In the surface layer, while the temperature in the BR (22.9 °C) was slightly lower than in the NR (23.5 °C), the BR exhibited dramatically elevated concentrations of NH_4_^+^-N (851.8 mg/L vs. NR’s 130.2 mg/L) and PO_4_^3−^-P (72.8 mg/L vs. NR’s 27.2 mg/L), representing 6.5-fold and 2.7-fold increases, respectively, compared to the NR (Figure S1). In the bottom layer, the salinity (28.8) and NH_4_^+^-N (222.0 mg/L) in the BR were significantly lower than that in the NR (29.6 and 311.1 mg/L), while the NO_3_^−^-N (147.3 mg/L) and NO_2_^−^-N (37.3 mg/L) in the BR were significantly higher than that in the NR (NO_3_^−^-N: 90.9 mg/L, NO_2_^−^-N: 21.4 mg/L).

### 3.2. Microbial Community Structure and Diversity

#### 3.2.1. Microbial Community Composition

A total of 8310 prokaryotic ASVs and 1892 microeukaryotic ASVs were identified. The total number of shared prokaryotic and microeukaryotic ASVs between the BR and the NR were 1506 and 565, respectively, constituting 18.1% and 29.9% of the total prokaryotic and microeukaryotic ASVs (Figure S2).

**Figure 3:**
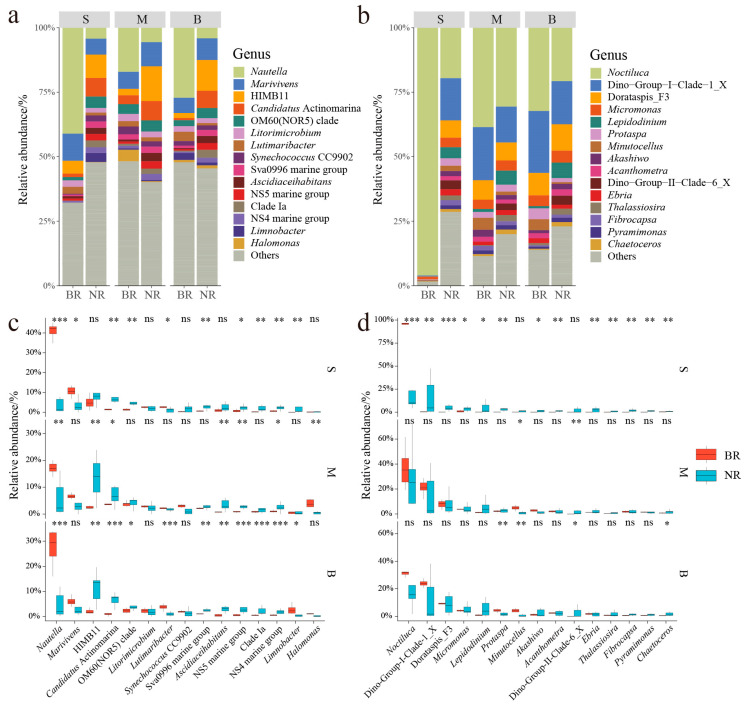
Taxonomic compositions of microbial communities in the blooming region (BR) and the non-blooming region (NR). (**a**) Relative abundances of genera of prokaryotes; (**b**) Relative abundances of genera of microeukaryotes; (**c**) Variance of the top 15 genera of prokaryotes; (**d**) Variations in the top 15 genera of microeukaryotes. S: surface layer, M: middle layer, B: bottom layer. Statistical significance of differences (***: *p* < 0.001, **: *p* < 0.01, *: *p* < 0.05, ns: *p* > 0.05) is determined by the Wilcoxon rank-sum test.

The prokaryotic communities in the BR and NR were primarily composed of the genera *Nautella*, *Marivivens*, HIMB11, *Candidatus* Actinomarina, OM60 (NOR5) clade, and *Litorimicrobium* (Figure 3a). Notably, the composition of these dominant genera exhibited considerable variation both between the regions and with respect to depth. In the BR, the surface layer was predominantly occupied by *Nautella* (41.04%), followed by *Marivivens* (10.50%) and HIMB11 (5.01%). The middle layer was dominated by *Nautella* (17.06%), *Marivivens* (6.64%) and *Halomonas* (4.56%), while in the bottom layer, the most abundant genera were *Nautella* (27.11%), *Marivivens* (5.92%), and *Lutimaribacter* (3.61%). In contrast, the surface layer in the NR was predominantly composed of HIMB11 (9.02%), Ca. Actinomarina (7.26%), and *Marivivens* (6.18%). The middle layer was similarly dominated by HIMB11 (13.48%), *Marivivens* (9.37%), and Ca. Actinomarina (7.52%), while the bottom layer was dominated by HIMB11 (11.91%), *Marivivens* (8.41%), and Ca. Actinomarina (6.69%). The differences in species composition between the two regions were evident in both the surface and bottom layers, with the most pronounced variation observed in the surface layer; however, no significant differences were noted in the middle layer. Importantly, the relative abundances of *Nautella* and *Marivivens* were significantly higher in the surface and bottom layers of the BR when compared to the NR. In contrast, Ca. Actinomarina and OM60 (NOR5) clade exhibited significantly lower relative abundances across all layers in the BR compared to the NR. Additionally, HIMB11 displayed a significantly lower relative abundance in the middle and bottom layers of the BR in comparison to the NR (Figure 3a,c).

The microeukaryotic communities in the BR and NR were predominantly composed of *Noctiluca*, Dino-Group-I-Clade-1_X, Dorataspis_F3, *Micromonas*, *Lepidodinium*, and *Protaspa* (Figure 3b). The composition of these dominant microeukaryotic genera exhibited considerable variation between the BR and NR regions as well as with respect to depth. In the surface layer of the BR, *Noctiluca* was overwhelmingly dominant, accounting for 96.02%. In the middle layer, *Noctiluca* remained significant at 38.52%, followed by Dino-Group-I-Clade-1_X (20.60%) and *Dorataspis*_F3 (7.58%). The bottom layer of the BR was dominated by *Noctiluca* (32.27%), Dino-Group-I-Clade-1_X (24.06%) and *Dorataspis*_F3 (8.66%). In contrast, the surface layer of the NR was dominated by *Noctiluca* (19.55%), Dino-Group-I-Clade-1_X (16.37%), and *Dorataspis*_F3 (6.72%). The middle layer of the NR exhibited similar trends, with *Noctiluca* (30.56%), Dino-Group-I-Clade-1_X (13.96%), and *Dorataspis*_F3 (6.96%) as the primary genera. The bottom layer of NR was also characterized by *Noctiluca* (20.69%), Dino-Group-I-Clade-1_X (16.74%), and *Dorataspis*_F3 (10.24%). The species composition in the middle and bottom layers showed no significant difference between the two regions; however, the primary variation was observed in the surface layer. *Noctiluca* displayed a markedly higher relative abundance in the BR compared to the NR, whereas Dino-Group-I-Clade-1_X, *Dorataspis*_F3, and *Micromonas* exhibited significantly lower relative abundances in the BR. Additionally, *Minutocellus* had a significantly higher relative abundance in both the middle and bottom layers of the BR compared to the NR, although no significant difference was observed in the surface layer (Figure 3b,d).

#### 3.2.2. Microbial Diversity

**Figure 4:**
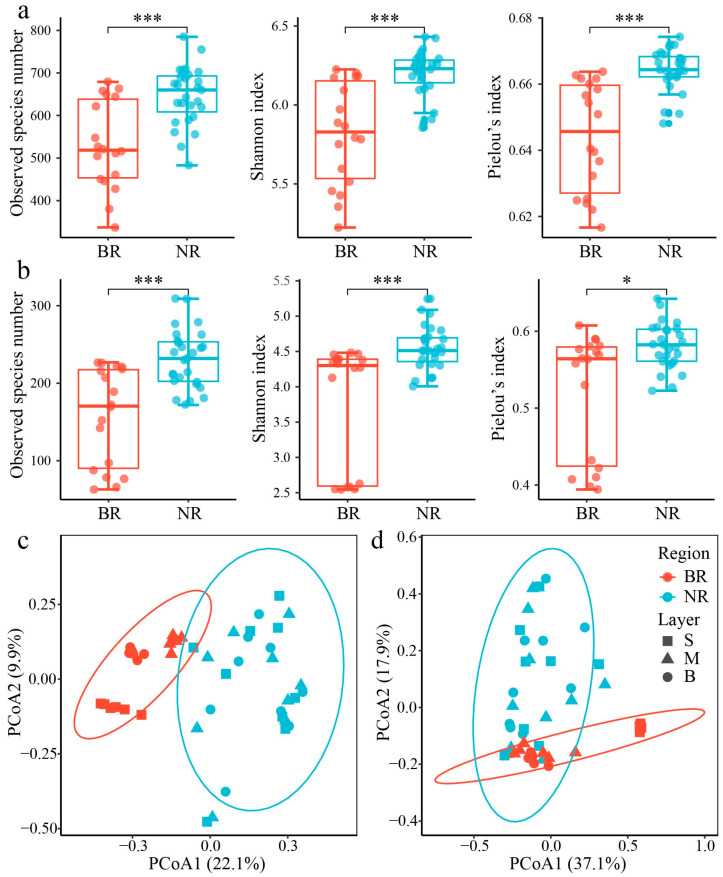
Comparison of microbial α-diversity and community composition between the blooming region (BR) and the non-blooming region (NR). (**a**,**b**) Boxplots of Sobs, Shannon, and Pielou’s indices for prokaryotic and microeukaryotic communities. Statistical significance of differences (***: *p* < 0.001, *: *p* < 0.05) is determined by the Wilcoxon rank-sum test. (**c**,**d**) Principal Coordinates Analysis (PCoA) illustrating the prokaryotic and microeukaryotic community dissimilarity. S: surface layer, M: middle layer, B: bottom layer.

Both prokaryotic and microeukaryotic communities exhibited significant differences in α-diversity between the NR and BR (Figure 4a,b), with all indices (Sobs, Shannon, and Pielou’s) consistently lower in the BR (*p* < 0.05). PCoA analysis further confirmed distinct community compositions (PERMANOVA: *p* = 0.001, Figure 4c,d), though the explanatory power of the first two axes were limited for both groups (prokaryotes: 32.0%, microeukaryotes: 55.0%), suggesting their community structures are more dimensionally complex between these regions. Notably, the BR surface communities (both prokaryotic and microeukaryotic) showed particularly significant dissimilarities with the NR communities.

### 3.3. Relationship Between Microbial Community Composition and Environmental Factors

**Figure 5:**
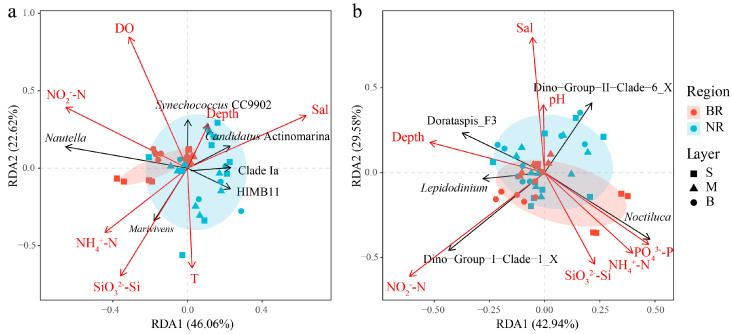
Relationship between the prokaryotic (**a**) and microeukaryotic (**b**) community compositions at the genus level and environmental variables. Abbreviations for layers and environmental variables are defined in Table 1.

The FS analysis indicated that the key environmental factors that significantly influenced the prokaryotic community structure were NO_2_^−^-N, SiO_3_^2−^-Si, DO, depth, salinity, NH_4_^+^-N, and temperature. They collectively explained 43.3% of the variation in prokaryotic community composition. On the RDA plot, most samples from the BR were concentrated in the second and third quadrants, which represented higher nutrient levels (Figure 5a). NO_2_^−^-N exhibited a positive correlation with *Nautella* and *Synechococcus* CC9902, while showing a negative correlation with HIMB11. Additionally, SiO_3_^2−^-Si was positively correlated with *Marivivens* and negatively correlated with *Synechococcus* CC9902.

The key environmental factors that significantly influenced the microeukaryotic community structure were NO_2_^−^-N, PO_4_^3−^-P, SiO_3_^2−^-Si, NH_4_^+^-N, depth, pH, and salinity. Collectively, these factors accounted for 47.5% of the variation in microeukaryotic community composition. Most samples from the BR were situated in the third and fourth quadrants of the RDA plot, indicative of higher nutrient levels (Figure 5b). The concentration of NO_2_^−^-N showed a positive correlation with Dino-Group-I-Clade-1_X and a negative correlation with Dino-Group-II-Clade-6_X. The concentration of PO_4_^3−^-P was positively correlated with *Noctiluca* and negatively correlated with *Dorataspis*_F3 and *Lepidodinium*. RDA further indicated that the relative abundance of *Noctiluca* was positively correlated with PO_4_^3−^-P, NH_4_^+^-N and SiO_3_^2−^-Si, while negatively correlated with NO_2_^−^-N, depth, salinity, and pH. In contrast, the Spearman correlation analysis revealed that the relative abundance of *N. scintillans* was significantly correlated only with NH_4_^+^-N (*p* < 0.05) (Figure S3).

### 3.4. Microbial Co-Occurrence Network

#### 3.4.1. Network Topological Properties

**Figure 6:**
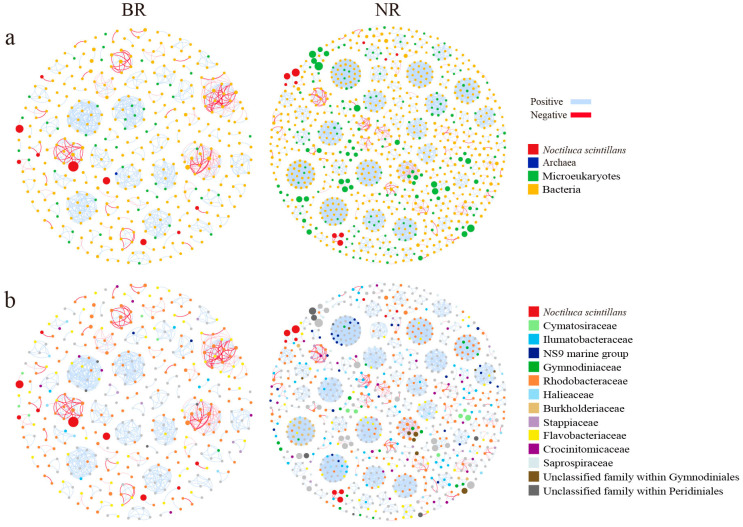
Comparison of the integrated co-occurrence networks of prokaryotes and microeukaryotes between the blooming region (BR) and the non-blooming region (NR). Panels (**a**,**b**) classify the nodes by color at the domain level and the class level, respectively. Nodes indicate individual ASVs and edges indicate significant correlations. The size of each node corresponds to the abundance of ASV, while the thickness of the connecting edges represents the strength of the correlation.

By comparing the topological properties of the integrated networks of prokaryotes and microeukaryotes between the BR and NR, we found that the BR network exhibited lower complexity and stability compared to the NR network. Specifically, the BR network had significantly fewer nodes, edges, and modules, as well as a lower average degree and clustering coefficient than the NR network. In contrast, the average path length and betweenness centrality were considerably higher in the BR network (Table 2, Figure 6). While the BR network contained a higher proportion of prokaryotic nodes (79.1%) compared to the NR network (94.1%), it exhibited a lower proportion of positive correlations. Furthermore, both the integrated networks in the BR and NR exhibited modularity greater than 0.4, confirming their multi-module structures (Table 2). When examining the independent prokaryotic and microeukaryotic networks across the regions, we found that the BR networks exhibited significantly fewer nodes, edges, and modules, and a lower average degree compared to the NR networks (Table S2, Figure S4). The BR prokaryotic network showed fewer positive correlations (77.0% vs. 94.3%), while the microeukaryotic networks exhibited minimal variation (98.6% vs. 99.3%). Notably, all networks maintained modularity greater than 0.4, further confirming their multi-module structures (Table S2).

#### 3.4.2. Network Keystone Taxa

In the integrated networks, the keystone ASVs with the highest betweenness centrality in the BR were mostly from Flavobacteriaceae and Rhodobacteraceae, while those in the NR were from a more diverse range of taxa, including Rhodobacteraceae, Cryptomonadales, Verrucomicrobiales, Gymnodiniaceae, Cryomonadida, Chlorarachniophyceae, Ilumatobacteraceae, Rubritaleaceae, and Actinomarinaceae (Table 3). In the independent prokaryotic networks, the keystone ASVs in the BR were exclusively from Flavobacteriaceae and Rhodobacteraceae. In contrast, the keystone ASVs in the NR were from diverse taxa, such as Verrucomicrobiales, Rubritaleaceae, Rhodobacteraceae, and Crocinitomicaceae (Table S3). In the independent microeukaryotic networks, all nodes in the BR had a betweenness centrality of zero, indicating a lack of keystone nodes. In contrast, in the NR, only three ASVs (belonging to Cryomonadida and Cymatosiraceae) had non-zero betweenness centrality and were identified as keystone taxa (Table S3).

#### 3.4.3. Network Modules

**Figure 7:**
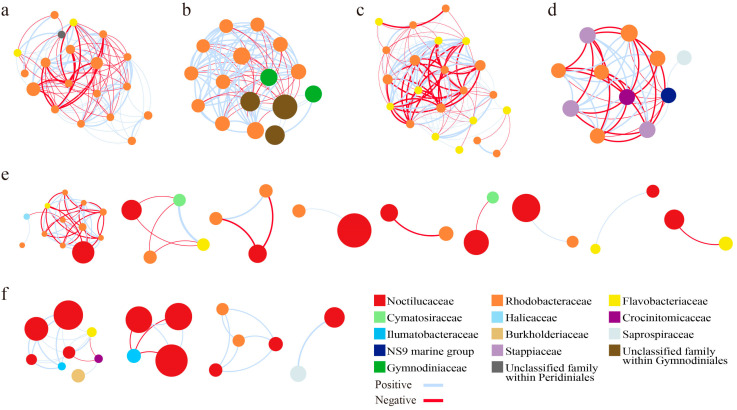
Comparison of important modules derived from the integrated networks of the blooming region (BR) and the non-blooming region (NR). (**a**) Largest module containing negative correlations from the BR network; (**b**) Largest module containing negative correlations from the NR network; (**c**) Largest module comprised entirely of prokaryotes with negative correlations from the BR network; (**d**) Largest module comprised entirely of prokaryotes with negative correlations from the NR network; (**e**) Modules associated with *Noctiluca scintillans* from the BR network; (**f**) Modules associated with *N. scintillans* from the NR network.

The main modules within the integrated networks of the BR and the NR exhibited significant differences. In the largest module containing negative correlations, the only microeukaryotic node in the BR network was from Peridiniales, whereas the microeukaryotic nodes in the NR network were predominantly from the family Gymnodiniaceae and other unclassified families within Gymnodiniales (Figure 7a,b). It was worth noting that as many as 33 nodes in the NR network were from Gymnodiniales, while only 1 node in the BR network was from Gymnodiniales. Regarding the largest module comprised entirely of prokaryotes with negative correlations, the nodes in the BR network were exclusively from Flavobacteriaceae and Rhodobacteraceae (Figure 7c). In contrast, the nodes in the NR network exhibited greater diversity, including members from Rhodobacteraceae, Stappiaceae, Crocinitomicaceae, and the NS9 marine group (Figure 7d). When considering the modules associated with *N. scintillans*, both the number of modules and the number of modules containing negative correlations in the BR network were notably greater than those in the NR network (Figure 7e,f). The nodes connected to *N. scintillans* in the BR network were notably less diverse, mostly belonging to Flavobacteriaceae and Rhodobacteraceae, while those in the NR network belonged to a broader range of taxa, i.e., Burkholderiaceae, Flavobacteriaceae, Crocinitomicaceae, Ilumatobacteraceae, Rhodobacteraceae, and Saprospiraceae (Figure 7e,f). It was noteworthy that in the BR network, the only microeukaryotic node that exhibited a significant negative correlation with *N. scintillans* was the ASVs from *Minutocellus* within Cymatosiraceae, whereas there were no microeukaryotic nodes significantly correlated with *N. scintillans* in the NR network (Figure 7e,f). Additionally, by the abovementioned comparisons, it was found that multiple Rhodobacteraceae ASVs were involved in these modules in both regions, while Flavobacteriaceae ASVs were more prevalent in the BR network (Figure 7).

### 3.5. Microbial Metabolic Functions

**Figure 8:**
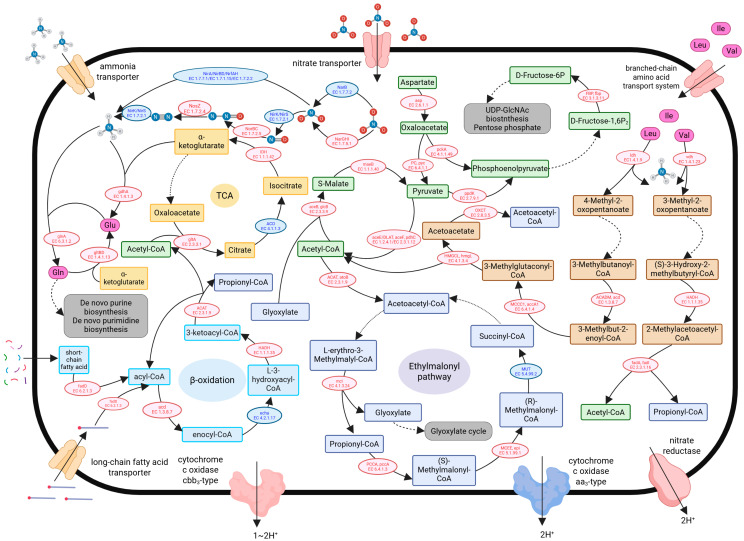
Schematic diagram of metabolic differences in the blooming region (BR) microbial community relative to the non-blooming region (NR). Solid arrows represent single-step catalytic reactions showing significant changes between regions, while dashed arrows indicate reactions with no significant differences or non-key processes. Downstream metabolic fates appear in gray rounded rectangles, inorganic nitrogen compounds as structural formulas, and amino acids as pink ovals. Organic metabolites are represented by rounded rectangles, color-coded by pathway: yellow for TCA cycle, blue for β-oxidation, purple for methylmalonyl-CoA pathway, green for pyruvate metabolism and gluconeogenesis, and brown for branched-chain amino acids. Membrane transporters, catalytic enzymes and their corresponding encoding genes are colored red (upregulated), blue (downregulated) or yellow (unchanged) to reflect differential expression patterns.

Gene prediction analysis indicated that the microbial communities in the BR exhibited multi-level metabolic reorganization characteristics compared to those in the NR (Figure 8). All analyses in this section are based on gene-level prediction results, and references to specific enzymes herein refer to the encoding genes of those enzymes.

#### 3.5.1. Respiratory Chain

The genes for the aa3-type cytochrome c oxidase (aerobic type), including *coxA*, *coxB*, *coxC*, and *coxD*, were downregulated, while the genes for the cbb3-type cytochrome c oxidase (hypoxia-adaptive type), including *ccoN*, *ccoO*, *ccoP*, and *ccoQ*, were upregulated (Figure 8). Additionally, the genes encoding the alternative electron transfer pathway—nitrate reductase (*NarGHI*)—were also upregulated.

#### 3.5.2. Fatty Acid Degradation, Methylmalonyl-CoA Pathway, and TCA Cycle

In the metabolic process of fatty acid degradation, long-chain fatty acid-CoA ligase (EC 6.2.1.3), a key enzyme in the β-oxidation pathway, was found to be upregulated (Figure 8). This upregulation suggests an enhancement of the fatty acid degradation pathway, leading to increased production of acetyl-CoA and propionyl-CoA. At the same time, key enzymes in the methylmalonyl-CoA pathway, such as methylmalonyl-CoA/ethylmalonyl-CoA epimerase (EC 5.1.99.1) and the alpha subunit of propionyl-CoA carboxylase (EC 6.4.1.3), were upregulated. Furthermore, acetyl-CoA C-acetyltransferase (EC 2.3.1.9), which serves as a link between these two pathways, was significantly upregulated. This indicates that acetyl-CoA and propionyl-CoA produced through β-oxidation might enter the methylmalonyl-CoA pathway, thereby enhancing its functionality.

#### 3.5.3. Pyruvate Metabolism and Gluconeogenesis

In the pyruvate metabolic pathway, key enzymes such as malate synthetase (EC 2.3.3.9), malate dehydrogenase (decarboxylating, NADP+, EC 1.1.1.40), and the core components of the pyruvate dehydrogenase complex (EC 1.2.4.1, EC 2.3.1.12) were found to be upregulated (Figure 8). Additionally, the upregulation of gluconeogenic rate-limiting enzymes, including pyruvate carboxylase (EC 6.4.1.1), ATP-dependent phosphoenolpyruvate carboxykinase (EC 4.1.1.49), and fructose-1,6-diphosphatase (EC 3.1.3.11), indicates enhanced gluconeogenic flux with increased glucose production efficiency.

#### 3.5.4. Branched-Chain Amino Acid Metabolism

The genes encoding the components of the branched-chain amino acid transport system in the ABC transport family, including *livM*, *livK*, *livH*, *livG*, and *livF*, were significantly upregulated in the BR (Figure 8). This indicates an increased capacity for branched-chain amino acid transport by microorganisms in this region. Although there were no significant differences in the genes related to ammonia transport, their relative abundances were high in both regions (e.g., the *AmtB* gene: BR: 0.088%; NR: 0.086%), suggesting that the ability of microorganisms to actively acquire ammonia was comparable between the two regions. Additionally, branched-chain amino acid degradation was markedly upregulated in the BR, particularly highlighted by the upregulation of key enzymes unique to the leucine degradation pathway—leucine dehydrogenase (EC 1.4.1.9), isovaleryl-CoA dehydrogenase (EC 1.3.8.4), and 3-methylcrotonyl-CoA carboxylase alpha subunit (EC 6.4.1.4), as well as the enzyme specific to the valine degradation pathway—valine dehydrogenase (EC 1.4.1.23) and the key enzyme unique to isoleucine metabolism—3-hydroxyacyl-CoA dehydrogenase (EC 1.1.1.35). Furthermore, acyl-CoA dehydrogenase (EC 1.3.8.7), a key enzyme common to all branched-chain amino acid degradation pathways, was also upregulated. Enzymes that convert the metabolic products of branched-chain amino acids into acetyl-CoA, acetoacetate, and propionyl-CoA, such as 3-hydroxy-3-methylglutaryl-CoA lyase (EC 4.1.3.4) and 3-hydroxyisobutyryl-CoA dehydrogenase (EC 1.1.1.31), were also upregulated.

#### 3.5.5. Nitrogen Metabolism

In nitrogen metabolism, the pathways for ammonia production in the BR—reduction and nitrogen fixation—were significantly downregulated compared to the NR (Figure 8). This was reflected by the downregulation of nitrite reductase (EC 1.7.1.15, EC 1.7.7.1, EC 1.7.2.2), which catalyzes the reduction in nitrite to ammonia, and nitrogenase (EC 1.18.6.1), which catalyzes the conversion of nitrogen gas to ammonia. However, the nitrate reductase (EC 1.7.5.1; encoding genes *NarGHI*), which catalyzes the first step of nitrate reduction, was upregulated. The process of denitrification was overall active, as indicated by the upregulation of nitrate reductase (EC 1.7.5.1) (the same as the first step of nitrate reduction), nitric oxide reductase (EC 1.7.2.5), and nitrous oxide reductase (EC 1.7.2.4). Nevertheless, the nitrite reductase (NO-forming) (EC 1.7.2.1), which catalyzes the second step of denitrification, was downregulated. Ammonia assimilation in the BR was significantly upregulated, specifically indicated by the upregulation of glutamate dehydrogenase (NAD(P)+) (EC 1.4.1.3) and glutamine synthetase (EC 6.3.1.2). Isocitrate dehydrogenase (EC 1.1.1.42) in the TCA cycle in the BR was also upregulated, which is beneficial for providing sufficient α-ketoglutarate for ammonia assimilation.

## 4. Discussion

### 4.1. Relationship Between the Noctiluca scintillans Bloom and Environmental Conditions

In this study, it was found that the relative abundance of *N. scintillans* was much higher in the surface layer than in the bottom layer, which was consistent with the results of previous studies on the spatial pattern of *N. scintillans* [24,35]. This is, on one hand, due to the characteristics of the *N. scintillans* cells, which contain a large ammonia-filled vesicle inside, making them less dense and more buoyant, and they can release large amounts of mucus, making it easier for the cells to aggregate [36,37]. On the other hand, it might be attributed to the fact that the tidal and current patterns in the coastal waters of Qinhuangdao facilitated the congregation of *N. scintillans* in the surface layers [24].

It is generally believed that the outbreak of HABs is related to the combined effects of suitable physical, chemical, and hydrometeorological factors, and the intensity and dynamics of which can also cause the occurrence of *N. scintillans* blooms [38]. In this study, we found that the physicochemical properties of seawater between the BR and the NR exhibited significant differences in both the surface and bottom layers, whereas the difference in the middle layer was not pronounced. Although the surface temperature in the BR was significantly lower than that in the NR, the Spearman correlation test showed that the correlation between the relative abundance of *N. scintillans* and temperature was not significant. This was due to the temperatures in both regions being within the general temperature range for *N. scintillans* blooms (18.0–25.0 °C) [25], and thus temperature was not the primary factor responsible for the formation of *N. scintillans* blooms.

The levels of PO_4_^3−^-P and NH_4_^+^-N in the surface waters of the BR, as well as the concentrations of NO_2_^−^-N and NO_3_^−^-N in the bottom waters of the BR, were significantly higher compared to those in the NR. Spearman correlation analysis revealed a significant positive correlation between the relative abundance of *N. scintillans* and NH_4_^+^-N concentration. Previous studies have shown that *N. scintillans* blooms are often associated with elevated levels of dissolved inorganic nutrients, including inorganic nitrogen, active phosphates, and soluble silicates, along with increased concentrations of trace elements [39]. This relationship may stem from abundant nutrients that support the growth of photosynthetic autotrophs, such as diatoms, which in turn provide ample prey for *N. scintillans*, facilitating its growth and reproduction [39]. Another scenario occurs when *N. scintillans* blooms transition to the diffusion phase; as many algal cells age and die, intracellular nutrients and organic matter are released into the seawater through cell lysis, leading to fluctuations in nutrient levels within the marine environment [40,41,42]. The nutrient vertical profiles showed that the elevated levels of PO_4_^3−^-P and NH_4_^+^-N were confined to the surface waters of the BR and were not observed at adjacent sites closer to shore. Therefore, the localized high nutrient levels likely result from nutrient release by *N. scintillans* cells rather than from terrestrial runoff or upwelling. The substantial accumulation of NH_4_^+^-N within the vesicles of *N. scintillans* ruptures during cellular senescence, releasing nitrogenous compounds such as ammonium, nitrate, and urea into the seawater [37]. Urea can subsequently be converted to ammonium through microbial metabolism, further elevating ammonium levels in the environment [43]. Similarly, the increase in phosphate may be attributed to cell lysis, which releases polyphosphate and organic phosphorus, thereby temporarily boosting bioavailable phosphate.

### 4.2. Impacts of the N. scintillans Bloom on Prokaryotic and Microeukaryotic Community Structure

It was observed that the α-diversity indices of both prokaryotic and microeukaryotic communities were significantly lower in the BR compared to the NR. This decrease was primarily due to the overwhelming dominance of *N. scintillans* in the BR, which suppressed the growth of other microeukaryotes and simultaneously triggered a rapid increase in the proportions of specific prokaryotes, allowing them to achieve absolute dominance. Results of PERMANOVA and the Wilcoxon rank-sum test indicated significant differences in the compositions of prokaryotic and microeukaryotic communities between the BR and NR, particularly in the surface waters. The dominant prokaryotic taxa that were significantly higher in the surface waters of the BR compared to the NR included *Nautella*, *Marivivens* and *Lutimaribacter*, which were all from the Rhodobacteraceae family. Rhodobacteriaceae are known to increase in abundance during phytoplankton blooms, as they consume freshly synthesized organic matter and are often associated with particles [44,45,46,47]. Moreover, *N. scintillans* hosts a significant proportion of endocytic Rhodobacteriaceae [45].

According to the results of FS, both prokaryotic and microeukaryotic community compositions exhibited significant correlations with various environmental factors. The variation in prokaryotic community composition was significantly correlated with factors including NO_2_^−^-N, SiO_3_^2−^-Si, DO, depth, salinity, NH_4_^+^-N and temperature. In contrast, the microeukaryotic community composition showed significant correlations with NO_2_^−^-N, PO_4_^3−^-P, SiO_3_^2−^-Si, NH_4_^+^-N, depth, pH and salinity. It was evident that nutrient levels were closely linked to both the prokaryotic and microeukaryotic community structures. Among the dominant microeukaryotic taxa, Dino-Group-I-Clade-1_X, which belongs to the parasitic Syndiniales, showed a positive correlation with NO_2_^−^-N. This lineage exhibited high relative abundance in the middle and bottom waters of the BR, likely due to the relatively high abundance of its host dinoflagellates in those same layers, where nutrient levels were sufficient, though *N. scintillans* were less abundant compared to the surface layer. Among the dominant prokaryotic taxa, those with higher relative abundances in the surface waters of the BR, such as *Nautella* and *Marivivens*, exhibited positive correlations with NO_2_^−^-N and SiO_3_^2−^-Si, respectively. Notably, the distribution of *Nautella* differed from that of *Marivivens*, as *Nautella* also showed higher relative abundances in the middle and bottom waters of the BR. Their distributions were presumably related primarily to the availability of organic matter they feed on, while their correlations with specific nutrients may be attributed to the collinearity between these nutrients and distinct types of organic matter.

### 4.3. Impacts of the N. scintillans Bloom on Microbial Interactions

Microbial co-occurrence networks can be applied to elucidate the complexity and interactions within microbial communities [48]. Various network topological parameters can reflect the network complexity, and the higher the network complexity, the higher the stability of the microbial network and the stronger the ability to adapt to changes in the external environment [49]. In this study, we analyzed integrated co-occurrence networks of prokaryotes and microeukaryotes, revealing that the number of nodes, edges, and the average degree of the BR network were significantly lower than those of the NR network. This finding indicated that the BR network exhibited lower complexity compared to the NR network. Furthermore, the average path length and betweenness centrality were higher in the BR network, while the number of modules was fewer. These characteristics suggested that the BR microbial network depended predominantly on nodes with higher betweenness centrality, demonstrating reduced connectivity and a lack of redundant pathways [50]. Consequently, if specific modules within the BR network were disrupted, it would be challenging to maintain functionality through alternative pathways, emphasizing the inherent vulnerability of the BR network and its lower stability. In contrast, the microbial communities in the NR exhibit a more intricate and stable network structure. Analysis of independent prokaryotic networks further confirmed that the complexity of the microbial network in the BR was noticeably lower than that in the NR. These findings indicate that the observed bloom of *N. scintillans* reduced the complexity and stability of the microbial network. However, HABs do not universally reduce the complexity of microbial networks; some studies suggest that the complexity may actually increase during HABs [51,52]. These contrasting effects likely reflect the distinct food web structures associated with different algal bloom scenarios.

Nodes with high betweenness centrality are often identified as keystone taxa, which play an important role in maintaining community stability [53]. Keystone taxa exert considerable influence on community composition, and their absence can result in substantial losses of community members [54]. Depending on the interaction topology and type (beneficial/antagonistic), keystone species can either promote or reduce species richness. In the BR network, the nodes exhibiting the highest betweenness centrality were predominantly from Flavobacteriaceae and Rhodobacteraceae, indicating their crucial contribution to network complexity and stability. Flavobacteriaceae can efficiently convert high molecular weight (HMW) compounds derived from phytoplankton, such as amino acids, organic acids, carbohydrates, and sugar alcohols, into low molecular weight (LMW) substances, including polysaccharides, proteins, nucleic acids, and lipids [46,55]. Rhodobacteriaceae are generally capable of degrading LMW carbohydrates and are thought to capitalize on partial degradation of algal exudates by other bacteria in the phycosphere [55]. Our results showed that the keystone taxa in the BR network included ASVs from the genus *Aurantivirga* within Flavobacteriaceae, as well as from HIMB11 and *Donghicola* within Rhodobacteraceae. *Aurantivirga* is recognized as one of the earliest responders to phytoplankton blooms, exhibiting a copiotrophic lifestyle characterized by rapid cycles of proliferation and decline. This dynamic is facilitated by its ability to outcompete other taxa through the digestion of algae-derived polysaccharides [56]. HIMB11 possesses genetic potential for degrading the algae-derived compound dimethysulfoniopropionate (DMSP), potentially increasing emissions of the gas dimethylsulfide (DMS) during *N. scintillans* blooms [45]. *Donghicola* is known to degrade bacteria-derived dissolved organic matter (DOM) and probably bacteria-derived lipids [57]. These taxa within Flavobacteriaceae and Rhodobacteraceae can synergistically remineralize larger components of phytoplankton organic matter [47]. The interplay among them, along with their distinct interactions with *N. scintillans*, played a crucial role in sustaining the stability of the integrated network during the bloom.

Positive correlations accounted for a proportion as high as 94.1% in the NR network, suggesting that the microorganisms have similar preferences and cooperative behaviors, i.e., cross-feeding, syntrophic interactions, and mutualistic interactions [58,59]. In contrast, in the BR network, although most of the correlations between the nodes were positive, there was also a certain proportion (20.9%) of negative correlations, which was much higher than that in the NR network. An increase in negative microbial correlations caused by algal blooms has also been described in other studies [60]. Negative correlations reflect antagonistic relationships among microorganisms, including competition, parasitism, and pathogenic relationships [53]. Among these antagonistic relationships, competitive relations may be the most prevalent, because the ecological niches of dominant microbial taxa in the BR were relatively similar and the utilization of similar resources intensified their competition.

In microbial co-occurrence networks, a module usually represents a group of microorganisms that interact strongly among themselves but little with taxa in other modules [50]. We compared the largest modules containing prokaryotes and microeukaryotes, the largest modules containing only prokaryotes, and the module associated with *N. scintillans* in the two regions. The nodes in the BR network were significantly less diverse at the family level, primarily belonging to Flavobacteriaceae and Rhodobacteraceae, whereas the nodes in the NR network belonged to more diverse families. Additionally, multiple Rhodobacteraceae ASVs were involved in these modules in both regions, while Flavobacteriaceae ASVs were more prevalent in the NR network. This indicated that the role of Flavobacteriaceae was significantly enhanced in the BR network compared to the NR network, due to their rapid response to the abundant algae-derived DOM produced during the bloom [46].

The number of modules associated with *N. scintillans* nodes in the BR network was greater than in the NR network, and a higher number of negative correlations were observed within these modules. Specifically, *N. scintillans* exhibited a negative correlation with the ASV from *Minutocellus* solely in the BR network. The genus *Minutocellus* showed a significantly higher relative abundance in the middle and bottom layers of the BR compared to the NR, while no significant difference was observed in the surface waters between the two regions. *Minutocellus* has been identified as a dominant symbiotic group associated with brown algae blooms caused by *Aureococcus anophagefferens* in the coastal waters of Qinhuangdao [61]. *Minutocellus* can grow and reproduce by utilizing nutrients such as nitrate, ammonia nitrogen, and organic nitrogen [62]. The observed negative correlation between *Minutocellus* and *N. scintillans*, along with the distribution characteristics of *Minutocellus* in the water column in the BR, might be attributed to the higher nutrient levels in the coastal waters of Qinhuangdao, which likely facilitated the reproduction of *Minutocellus*, thereby providing a nutritional basis for the bloom of *N. scintillans* [63]. However, this hypothesis remains speculative, and we expect that future studies conducted at multiple time points will provide further insights into the predation-prey relationships during HABs.

### 4.4. Impacts of the N. scintillans Bloom on Microbial Metabolism

The upregulation of hypoxia-adaptive cytochrome oxidase genes in the electron transport chain [64], along with the upregulation of nitrate reductase genes representing an alternative electron transfer pathway, indicates that in the bloom environment, the aerobic energy-production pathway in microorganisms might no longer be the sole dominant energy-generating process. Instead, anaerobic energy metabolism utilizing nitrate as an electron acceptor was enhanced [65,66]. A possible reason for this is that although the DO levels in the BR were not low, the high organic matter content could facilitate the aggregation of microbial communities, which leads to the formation of local hypoxic microenvironments characterized by a relative deficiency of electron acceptors, thereby promoting enhanced anaerobic metabolic processes.

The upregulation of key enzymes in the β-oxidation pathway suggests that this process might lead to an increased production of acetyl-CoA and propionyl-CoA. Additionally, the upregulation of key enzymes in the methylmalonyl-CoA pathway, along with those linking acetyl-CoA and propionyl-CoA to this pathway, indicates that acetyl-CoA and propionyl-CoA generated from β-oxidation could be utilized by the methylmalonyl-CoA pathway to convert into succinyl-CoA [67,68], which then enters the TCA cycle. This enhanced process facilitates the complete oxidation of unconventional carbon sources, such as odd-chain fatty acids, thereby maximizing energy utilization.

The upregulation of fatty acid degradation pathways could improve the efficiency of acetyl-CoA production. In the pyruvate metabolic pathway, the simultaneous enhancement of two consecutive steps—the conversion of acetyl-CoA to malate by malate synthase and the subsequent transformation of malate to pyruvate by malate dehydrogenase [69,70]—was likely linked to the availability of ample acetyl-CoA substrates. This sequential upregulation could facilitate the conversion of acetyl-CoA into pyruvate, thereby stimulating the gluconeogenic pathway that uses pyruvate as a substrate. The upregulation of multiple key enzymes in gluconeogenesis supports this hypothesis to a certain extent. The resulting fructose-6-phosphate might enter the biosynthetic pathway of UDP-N-acetylglucosamine and participate in the synthesis of materials that constitute cellular structures, such as glycosaminoglycans and ribose.

The upregulation of two specific enzymes involved in the deamination of leucine and valine, alongside a lack of significant changes in branched-chain amino acid transaminases, suggests that in addition to acquiring ammonia through ammonia transport proteins, there might exist a potential relationship whereby leucine or valine, under the catalysis of their specific deaminating enzymes [71,72], produce free ammonia that can be captured and utilized by glutamine synthetase or glutamate dehydrogenase. Moreover, the upregulation of enzymes converting branched-chain amino acid metabolic products into acetyl-CoA, acetoacetate, and propionyl-CoA indicates that the end products of branched-chain amino acid metabolism were being transformed into active intermediate metabolites [68]. These metabolites could be integrated into structural cellular components through processes such as gluconeogenesis or via the methylmalonyl pathway, ultimately entering energetic pathways such as the TCA cycle.

In nitrogen metabolism, the nitrite reductase that catalyzes the reduction in nitrite to ammonia and the nitrogenase that facilitates the conversion of nitrogen gas to ammonia were both downregulated in the BR. This might be due to the high concentration of ammonia, a product of these enzymatic reactions, which exerts a negative feedback regulation on the enzymes, leading to suppression of the ammonia production pathway [73,74]. Conversely, the gene for nitrate reductase, *NarGHI*, which catalyzes the initial step in nitrate reduction, was upregulated. This might be due to the low-oxygen or micro-oxic conditions created by high organic matter content in the BR [75]. Under these conditions, the limited availability of oxygen as an electron acceptor allowed nitrate to serve both as an electron acceptor and as a means to establish a transmembrane proton gradient, thereby supporting ATP synthesis through alternative electron transport pathways and compensating for energy production limitations in low-oxygen environments.

The process of denitrification appeared to be highly active in the BR, as indicated by the upregulation of nitrate reductase, nitric oxide reductase, and nitrous oxide reductase. This suggests that the primary fate of nitrate in this environment was denitrification, leading to the release of nitrogen gas from seawater rather than its reduction to ammonia. However, the downregulation of nitrite reductase (NO-type), which catalyzes the second step of the denitrification process, indicates that the efficiency of denitrification might be limited. This could be attributed to the suppression of enzyme expression in a micro-oxic environment [76].

Furthermore, the nitrogen assimilation processes in the BR were significantly upregulated, suggesting that microorganisms tended to convert ammonia into biologically available organic nitrogen. This is specifically shown by the upregulation of glutamate dehydrogenase and glutamine synthetase, where the former catalyzes the condensation of α-ketoglutarate with ammonia to form glutamate, and the latter catalyzes the condensation of glutamate with ammonia to form glutamine. These enzymatic processes are crucial pathways for microbial ammonia assimilation. Notably, α-ketoglutarate, a substrate for glutamate dehydrogenase, primarily originates from the tricarboxylic acid (TCA) cycle. The upregulation of isocitrate dehydrogenase in the TCA cycle within the BR ensured a sufficient supply of α-ketoglutarate, thereby maintaining the continuity of ammonia assimilation pathways. By converting ammonia into neutral, non-toxic glutamine, microorganisms might effectively alleviate ammonia toxicity in the high-ammonia environment of the BR, representing a key strategy for surviving in such conditions.

## 5. Conclusions

This study conducted a comparative analysis of the environmental conditions, microbial community structure, interactions, and metabolic features between the BR and NR, aiming for a better understanding of the causes and ecological impacts of *Noctiluca scintillans* blooms. Our findings revealed elevated inorganic nutrient levels in the BR, likely creating conditions favorable for the proliferation of autotrophic phytoplankton, such as *Minutocellus* spp., which ultimately led to the occurrence of the *N. scintillans* bloom.

This bloom significantly altered the community structure and diversity of prokaryotes and microeukaryotes in the pelagic environment. Specifically, the α-diversity indices for both prokaryotic and microeukaryotic communities were significantly lower in the BR compared to the NR. This decline was primarily due to the dominance of *N. scintillans*, which suppressed the growth of other microeukaryotes while simultaneously triggering a rapid increase in specific prokaryotic proportions. Furthermore, the bloom reduced the complexity and stability of the microbial co-occurrence network and significantly impacted the composition of keystone taxa. The keystone taxa within several complex modules of the BR network predominantly belonged to Rhodobacteriaceae and Flavobacteriaceae, suggesting that these taxa played crucial roles in maintaining network stability. In contrast, the taxa in the NR network exhibited greater diversity.

The *N. scintillans* bloom also induced a shift in microbial metabolic patterns. Despite ambient oxygen availability in the bloom environment, the microbial community transitioned from predominantly aerobic respiration to energy-yielding pathways adapted to micro-oxic/anoxic conditions. This shift likely resulted from localized oxygen depletion within micro-niches, driven by intense microbial competition for carbon sources in the high organic matter environment. Microbial communities in the BR optimized carbon substrate utilization by enhancing the degradation of fatty acids and branched-chain amino acids, channeling the resulting metabolites directly into the TCA cycle or via the methylmalonyl-CoA pathway for TCA cycle entry. While the TCA cycle was partially downregulated, these communities managed to maintain a balance between gluconeogenesis and energy production through flexible interconversion of pyruvate and malate. Additionally, in the nitrogen cycle, ammonium-mediated feedback inhibition suppressed ammonium-generating processes (e.g., nitrogen fixation), while denitrification pathways were significantly upregulated, becoming the dominant process for nitrogen transformation.

In summary, our research underscores the intricate relationships between nutrient enrichment, microbial community dynamics, and metabolic responses that collectively drive the ecological shifts associated with a *N. scintillans* bloom, highlighting the need for further investigation into the implications of these interactions on marine ecosystem health.

## Figures and Tables

**Table 1:** Comparison of parameters of seawater samples from the blooming region (BR) and the non-blooming region (NR) in the Qinhuangdao coastal waters.

Layer	T (°C)		Sal		Depth (m)		pH		DO (mg/L)		Chl-*a* (µg/L)		NO_3_^−^-N (mg/L)		NO_2_^−^-N (mg/L)		NH_4_^+^-N (mg/L)		PO_4_^3−^-P (mg/L)		SiO_3_^2−^-Si (mg/L)	
	BR	NR	BR	NR	BR	NR	BR	NR	BR	NR	BR	NR	BR	NR	BR	NR	BR	NR	BR	NR	BR	NR
S	22.9 *	23.5 *	29.1	29.1	0.2	0.1	7.7	7.6	7.5	7.1	2.7	2.1	184.4	240.8	29.7	28.9	851.8 *	130.2 *	72.8 *	27.2 *	447.9	260.4
M	22.8	22.9	29.6	29.5	5.6	5.1	7.7	7.6	7.4	7.1	2.5	2.1	96.5	97.3	24.5	21.0	291.5	316.1	13.7	10.8	395.0	574.4
B	22.7	22.9	28.8 **	29.6 **	11.1	10.0	7.7	7.6	7.6	7.1	3.0	3.1	147.3 *	90.9 *	37.3 **	21.4 **	222.0 *	**311.1**	51.6	12.4	516.7	502.2

S: surface layer; M: middle layer; B: bottom layer; T: temperature; Sal: salinity; DO: dissolved oxygen; Chl-*a*: chlorophyll *a*; NO_3_^−^-N: nitrate; NO_2_^−^-N: nitrite; NH_4_^+^-N: ammonium; PO_4_^3−^-P: phosphate; SiO_3_^2−^-Si: silicate. Environmental factors exhibiting significant variations (Wilcoxon rank-sum test: *p* < 0.05) are highlighted in bold, with statistical significance denoted as: **: *p* < 0.01, *: *p* < 0.05.

**Table 2:** Comparison of topological properties of the integrated co-occurrence networks of prokaryotes and microeukaryotes between the blooming region (BR) and the non-blooming region (NR).

Network Properties	BR	NR
Number of nodes	401	903
Number of prokaryotic nodes	342	697
Number of microeukaryotic nodes	59	206
Number of edges	1040	4300
Proportion of positive correlations	79.1%	94.1%
Average degree	5.187	9.524
Average path length	1.303	1.123
Network diameter	6	6
Clustering coefficient	0.953	0.995
Betweenness centrality	0.0005	0.0001
Number of modules	95	159
Modularity	0.943	0.946

**Table 3:** Comparison of the keystone taxa with the highest betweenness centrality between the blooming region (BR) and the non-blooming region (NR).

BR			NR		
Genus	Identifiable Taxonomic Level Above Genus	Betweenness Centrality	Genus	Identifiable Taxonomic Level Above Genus	Betweenness Centrality
*Aurantivirga*	Flavobacteriaceae	38	Unclassified	Rhodobacteraceae	42
HIMB11	Rhodobacteraceae	25.5	Unclassified	Cryptomonadales	30
HIMB11	Rhodobacteraceae	25.5	Unclassified	Verrucomicrobiales	24
*Donghicola*	Rhodobacteraceae	18	*Gymnodinium*	Gymnodiniaceae	24
*Aurantivirga*	Flavobacteriaceae	16	*Protaspa*	Cryomonadida	21
*Donghicola*	Rhodobacteraceae	16	*Marivivens*	Rhodobacteraceae	20
*Aurantivirga*	Flavobacteriaceae	16	Unclassified	Chlorarachniophyceae	20
Unclassified	Rhodobacteraceae	16	Unclassified	Ilumatobacteraceae	18
Sva0996 marine group	Microtrichaceae	15	*Roseibacillus*	Rubritaleaceae	18
HIMB11	Rhodobacteraceae	15	*Ca.* Actinomarina	Actinomarinaceae	17.5

## Data Availability

All the raw sequence files of this study were submitted to the National Center for Biotechnology Information (NCBI) with the study accession number PRJNA1187025. All data are available upon request to the authors.

## Supplementary Materials

The following supporting information can be downloaded at: https://www.mdpi.com/article/10.3390/microorganisms13081959/s1, Figure S1. Vertical distribution of the measured environmental factors. The star indicates the location of the blooming region (BR), and the red box highlights the vertical distribution of these factors within the BR. Figure S2. Venn diagram based on the prokaryotic (a) and microeukaryotic (b) ASVs in the blooming region (BR) and the non-blooming region (NR). Figure S3. Correlations between the relative abundance of *Noctiluca scintillans* and environmental factors. Figure S4. Comparison of the independent prokaryotic (a) and microeukaryotic (b) co-occurrence networks between the blooming region (BR) and the non-blooming region (NR). Nodes indicate individual ASVs and edges indicate significant correlations. The size of each node corresponds to the abundance of ASV, while the thickness of the connecting edges represents the strength of the correlation. Table S1. Depth of sampling layers. Table S2. Comparison of topological properties of the independent prokaryotic and microeukaryotic networks between the blooming region (BR) and the non-blooming region (NR). Table S3. Comparison of the keystone taxa with the highest betweenness centrality in independent prokaryotic and microeukaryotic networks between the blooming region (BR) and the non-blooming region (NR).
