# Supplementary material for: Noctiluca scintillans Bloom Reshapes Microbial Community Structure, Interaction Networks, and Metabolism Patterns in Qinhuangdao Coastal Waters, China
Source: Microorganisms. 2025 Aug 21;13(8):1959. doi: 10.3390/microorganisms13081959 (PMC12388393; doi:10.3390/microorganisms13081959)
Supplement: Supplementary file 1 [file microorganisms-13-01959-s001.zip › microorganisms-3798064-supplementary.pdf]

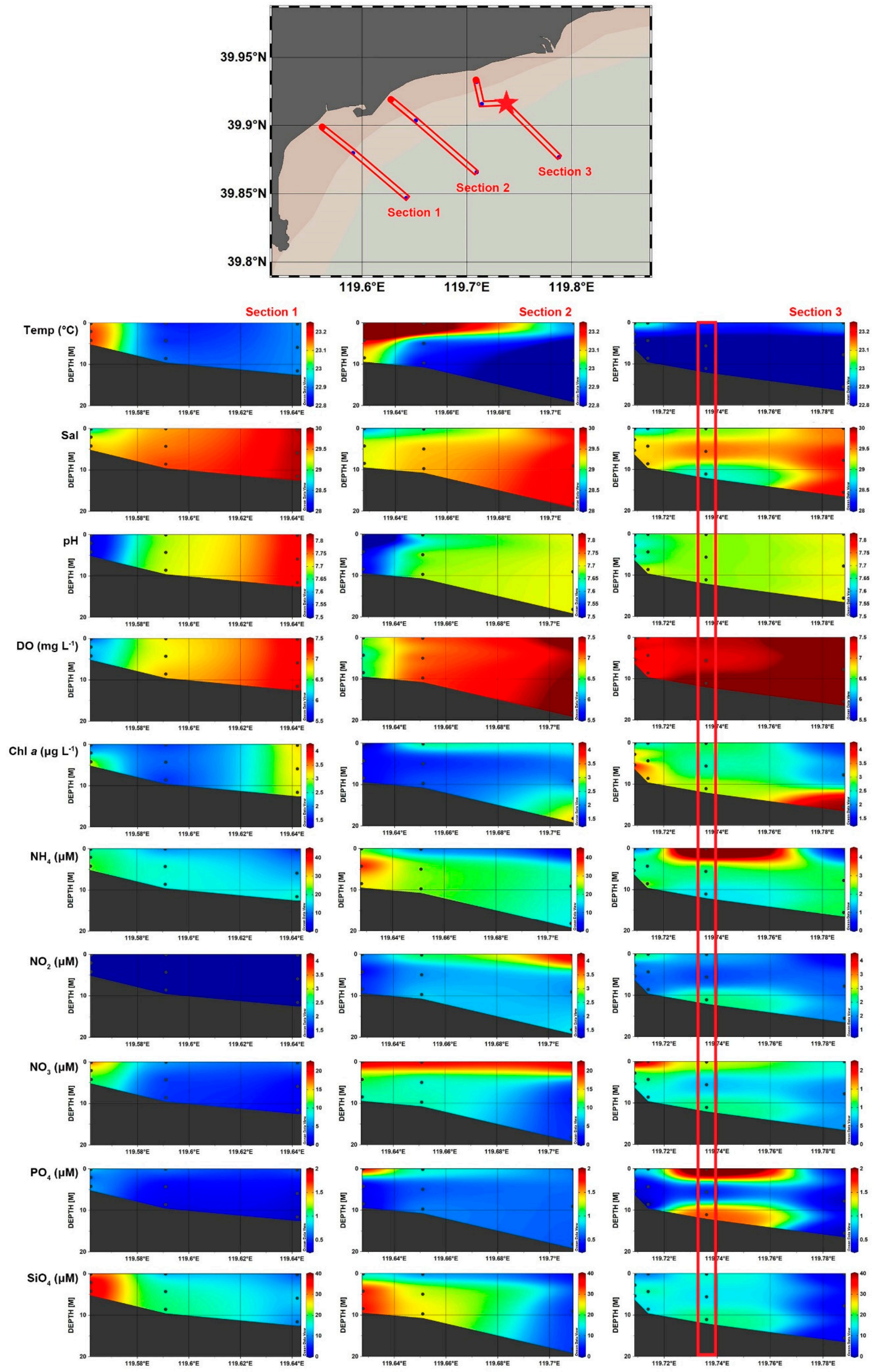

39.95°N
39.9°N
39.85°N
39.8°N
119.6°E
119.7°E
119.8°E
Section 1
Section 2
Section 3
Temp (°C)
Sal
pH
DO (mg L-1)
Chl a (μg L-1)
NH4 (μM)
NO2 (μM)
NO3 (μM)
PO4 (μM)
SiO4 (μM)
DEPTH [M]

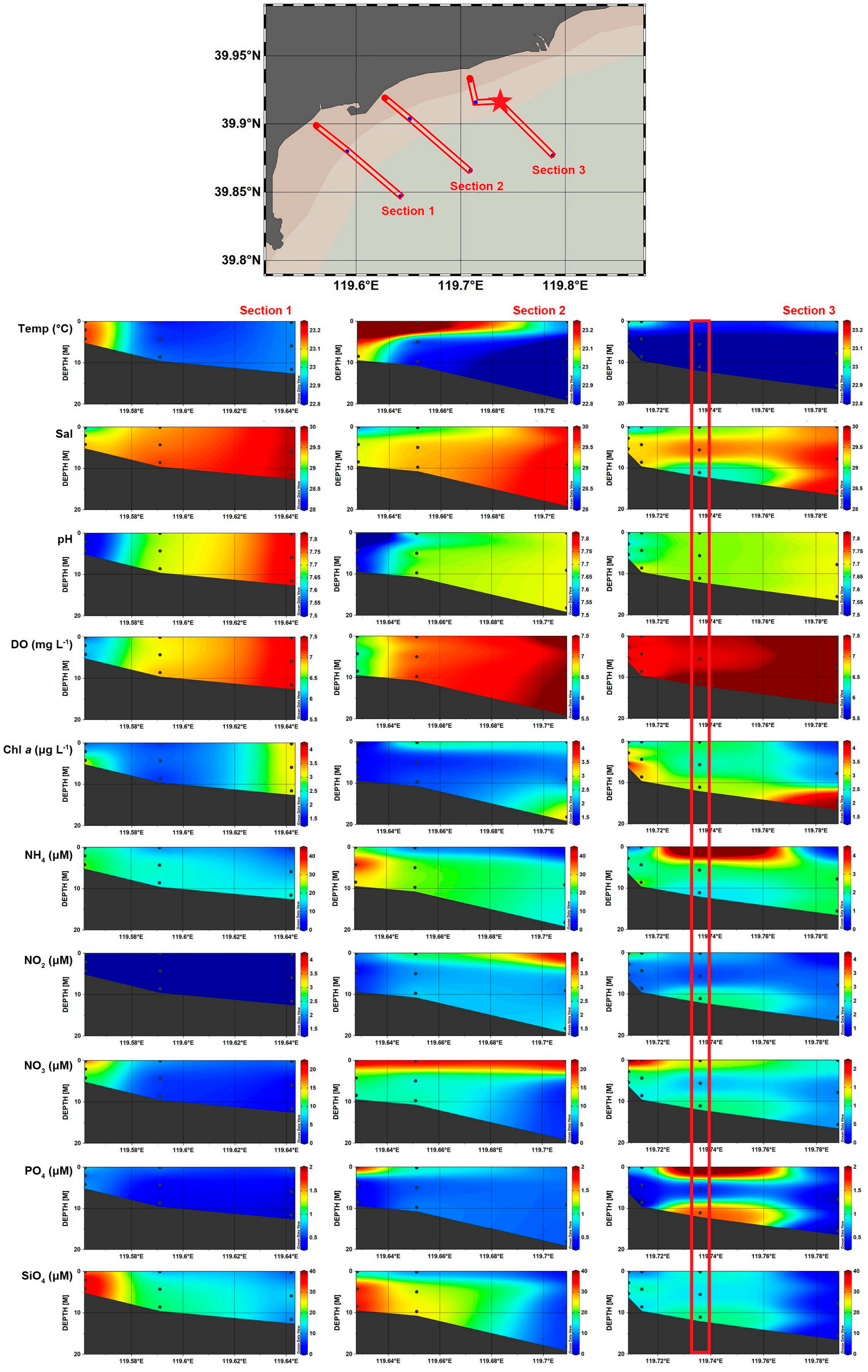
39.95°N
39.9°N
39.85°N
39.8°N
119.6°E
119.7°E
119.8°E
Section 1
Section 2
Section 3
Temp (°C)
Sal
pH
DO (mg L-1)
Chl a (μg L-1)
NH4 (μM)
NO2 (μM)
NO3 (μM)
PO4 (μM)
SiO4 (μM)
DEPTH [M]
119.58°E
119.6°E
119.62°E
119.64°E
119.66°E
119.68°E
119.7°E
119.72°E
119.74°E
119.76°E
119.78°E
Ocean Data View

Figure S1. Vertical distribution of the measured environmental factors. The star indicates the location of the blooming region (BR), and the red box highlights the vertical distribution of these factors within the BR.

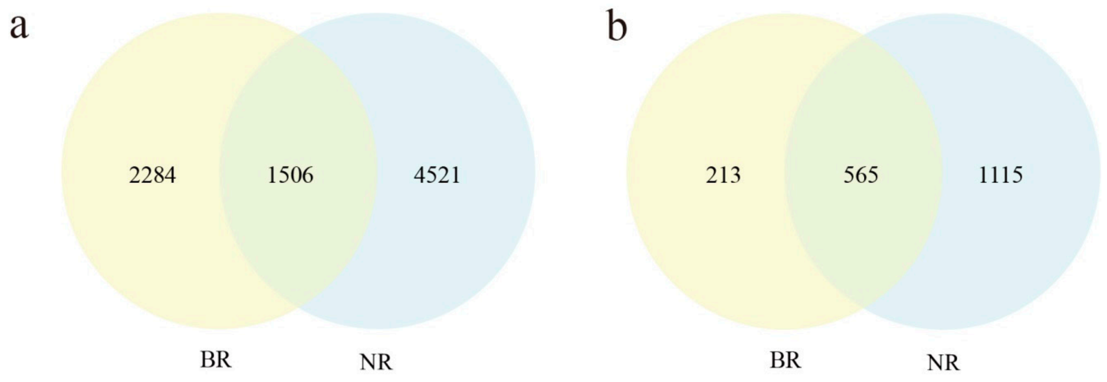

Figure S2. Venn diagram based on the prokaryotic (a) and microeukaryotic (b) ASVs in the blooming region (BR) and the non-blooming region (NR).

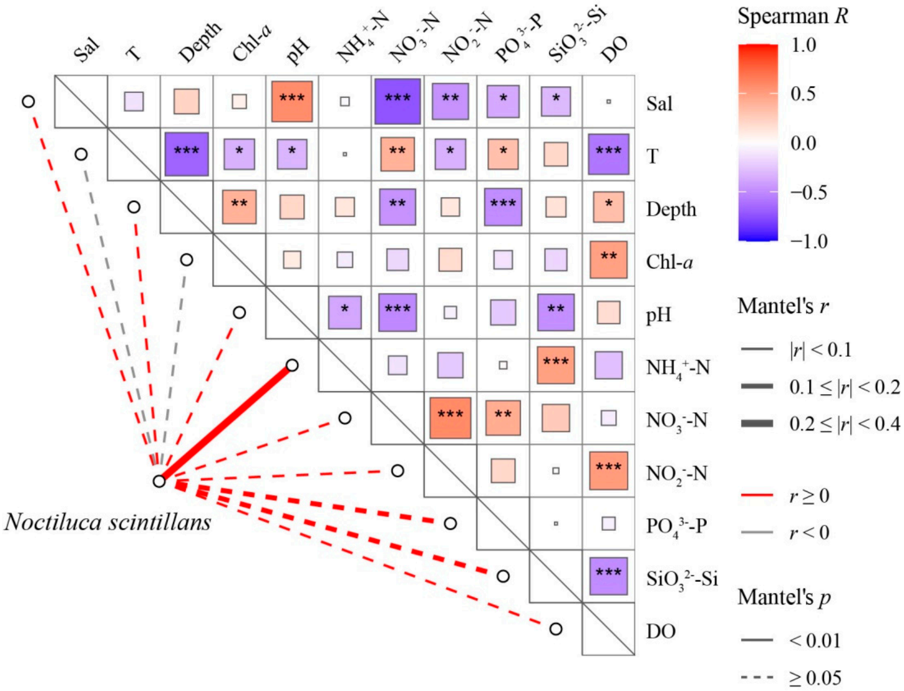

Figure S3. Correlations between the relative abundance of *Noctiluca scintillans* and environmental factors. The straight lines depict the Mantel correlation coefficients between *N. scintillans* and various environmental factors. The thickness of each line corresponds to the absolute magnitude of the correlation coefficient, while the color of the line represents the statistical significance of the correlation, with solid lines indicating positive correlations and dashed lines indicating negative correlations. Additionally, the Spearman correlation coefficients among the environmental factors are illustrated using a color scale ranging from -1 to 1. Asterisks denote levels of significance as follows: *** $P < 0.001$, ** $P < 0.01$, * $P < 0.05$, and blank indicates $P > 0.05$.

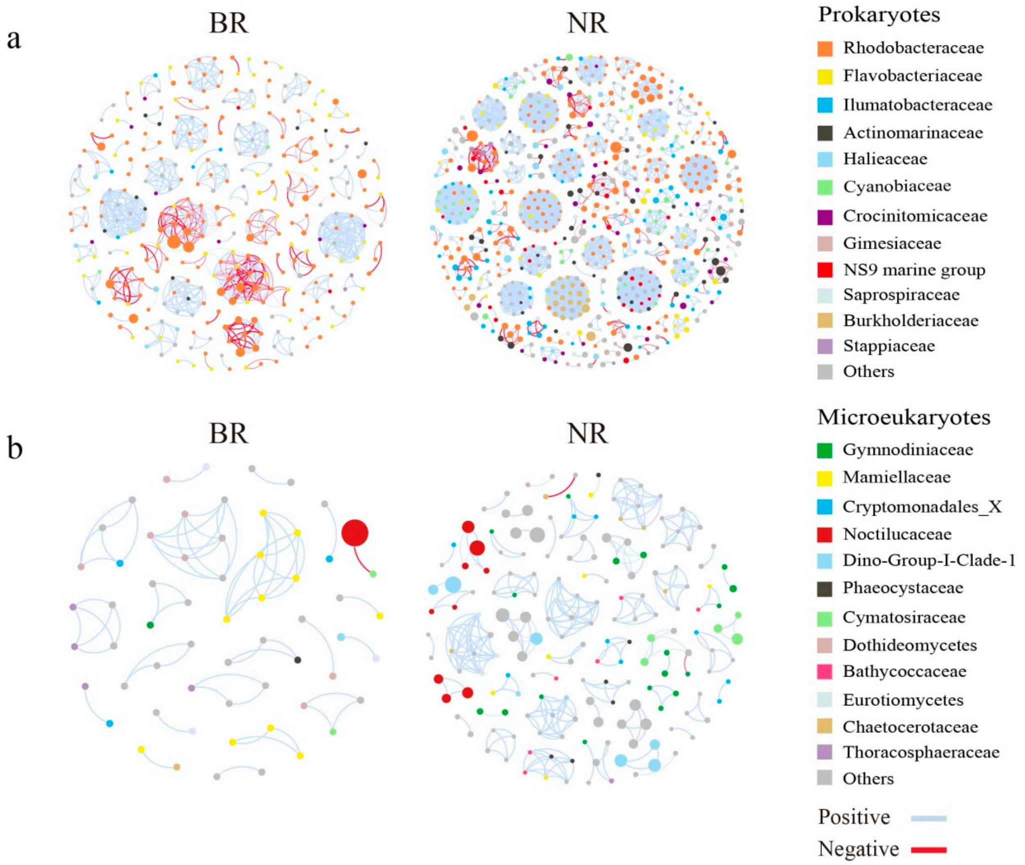

Figure S4. Comparison of the independent prokaryotic (a) and microeukaryotic (b) co-occurrence networks between the blooming region (BR) and the non-blooming region (NR). Nodes indicate individual ASVs and edges indicate significant correlations. The size of each node corresponds to the abundance of ASV, while the thickness of the connecting edges represents the strength of the correlation.

Table S1. Depth of sampling layers.

SampleID	Site	Sampling layer	Sampling depth (m)
NR1_S	NR1	Surface	0.1
NR1_M	NR1	Middle	2.8
NR1_B	NR1	Bottom	5.4
NR2_S	NR2	Surface	0.1
NR2_M	NR2	Middle	4.4
NR2_B	NR2	Bottom	8.6
NR3_S	NR3	Surface	0.1
NR3_M	NR3	Middle	7.8
NR3_B	NR3	Bottom	15.5
NR4_S	NR4	Surface	0.1
NR4_M	NR4	Middle	4.3
NR4_B	NR4	Bottom	8.5
NR5_S	NR5	Surface	0.2
NR5_M	NR5	Middle	5.0
NR5_B	NR5	Bottom	9.8
NR6_S	NR6	Surface	0.1
NR6_M	NR6	Middle	9.1
NR6_B	NR6	Bottom	18.2
NR7_S	NR7	Surface	0.1
NR7_M	NR7	Middle	2.1
NR7_B	NR7	Bottom	4.2
NR8_S	NR8	Surface	0.1
NR8_M	NR8	Middle	4.3
NR8_B	NR8	Bottom	8.6
NR9_S	NR9	Surface	0.2
NR9_M	NR9	Middle	5.9
NR9_B	NR9	Bottom	11.6
BR1_S	BR1	Surface	0.2
BR1_M	BR1	Middle	5.6
BR1_B	BR1	Bottom	11.1
BR2_S	BR2	Surface	0.2
BR2_M	BR2	Middle	5.6
BR2_B	BR2	Bottom	11.1
BR3_S	BR3	Surface	0.2
BR3_M	BR3	Middle	5.6
BR3_B	BR3	Bottom	11.1
BR4_S	BR4	Surface	0.2
BR4_M	BR4	Middle	5.6
BR4_B	BR4	Bottom	11.1
BR5_S	BR5	Surface	0.2

BR5_M	BR5	Middle	5.6
BR5_B	BR5	Bottom	11.1
BR6_S	BR6	Surface	0.2
BR6_M	BR6	Middle	5.6
BR6_B	BR6	Bottom	11.1

Table S2. Comparison of topological properties of the independent prokaryotic and microeukaryotic networks between the blooming region (BR) and the non-blooming region (NR).

Network properties	Prokaryotes		Microeukaryotes	
	**BR**	**NR**	**BR**	**NR**
Number of nodes	328	686	60	181
Number of edges	820	3028	71	292
Proportion of positive correlations	77.0%	94.3%	98.6%	99.3%
Average degree	5.000	8.828	2.367	3.227
Average path length	1.353	1.086	1	1.017
Network diameter	6	6	1	3
Clustering coefficient	0.947	0.997	1	0.996
Betweenness centrality	0.001	0.0002	0	0.0002
Number of modules	78	133	21	53
Modularity	0.933	0.939	0.884	0.949

Table S3. Comparison of the keystone taxa with the highest betweenness centrality in independent prokaryotic and microeukaryotic networks between the blooming region (BR) and the non-blooming region (NR).

Group	BR			NR		
	Genus	Identifiable taxonomic level above genus	Betweenness centrality	Genus	Identifiable taxonomic level above genus	Betweenness centrality
Prokaryotes	*Aurantivirga*	Flavobacteriaceae	60	Unclassified	Verrucomicrobiales	36
	Aurantivirga	Flavobacteriaceae	36	*Roseibacillus*	Rubritaleaceae	24
	HIMB11	Rhodobacteraceae	17	*Ascidiaceihabitans*	Rhodobacteraceae	21
	HIMB11	Rhodobacteraceae	17	Unclassified	Crocinitomicaceae	17.5
	Aurantivirga	Flavobacteriaceae	15	HIMB11	Rhodobacteraceae	17.5
	Donghicola	Rhodobacteraceae	15	Unclassified	Ilumatobacteraceae	16
	Aurantivirga	Flavobacteriaceae	15	*Marivivens*	Rhodobacteraceae	12
	Donghicola	Rhodobacteraceae	14	Unclassified	Flavobacteriales (NS9 marine group)	10
	Unclassified	Rhodobacteraceae	10	*Marivivens*	Rhodobacteraceae	10
	Marivivens	Rhodobacteraceae	8.5	Clade Ia	Alphaproteobacteria (SAR11 clade)	6
Microeukaryotes	—	—	—	*Protaspa*	Cryomonadida	3
	—	—	—	Unclassified	Cymatosiraceae	1
	—	—	—	*Minutocellus*	Cymatosiraceae	1
